# Supplementary material for: Semi-parametric model for timing of first childbirth after HIV diagnosis among women of childbearing age in Ibadan, Nigeria
Source: PLoS One. 2020 Oct 7;15(10):e0240247. doi: 10.1371/journal.pone.0240247 (PMC7540879; doi:10.1371/journal.pone.0240247)
Supplement: Attachment — Submitted filename: Response to Reviewers_R2edit.pdf [file pone.0240247.s002.pdf]

# DEPARTMENT OF EPIDEMIOLOGY AND MEDICAL STATISTICS

FACULTY OF PUBLIC HEALTH
COLLEGE OF MEDICINE
UNIVERSITY OF IBADAN, NIGERIA.

Email: ems@comui.edu.ng

Ag. Head: Dr. Magbagbeola D .DAIRO



ACADEMIC STAFF

EPIDEMIOLOGY

**Olufunmilayo I. Fawole, Professor**
MB;BS (Ib.) M.Sc. (Epid & Biost.) (SA), FMCPH (Nig), FWACP, Cert Clinical Epid. (Pretoria), F.Med.Ed. (SA)

**Ikeoluwapo O. Ajayi, Professor**
MB;BS (Ib.) M.CL.SC (Ontario), MPH (Comm. Health) (Ib), Ph.D, (Epid) (Ib), Cert. Field Epid. (USA),FMCGP (Nig), FWACP (Family Med.)

**M. D. Dairo, Reader**
MB;BS (Ib), M.Sc (Epid & Med. Stat.) (Ib.) M.Sc., (Bioethics) (Ib.) FMCPH (Nig.) F.Med.Ed (SA)

**B. O. Adedokun, Lecturer I**
MB;BS (Ib), M.Sc. (Epid. & Med. Stat.) (Ib), Ph.D (Ib)

**Ikeola A. Adeoye, Lecturer I**
MB;BS (Ife), MPH (Ife), FMCPH (Nig)

**E. A. Bamgboye, Lecturer I**
MB;BS (Ib), M.Sc. (Epid. & Med. Stat.) (Ib.), P.W.W.C.P. Cert. Clin. Epid (Rotterdam)

**S. Bello, Lecturer I**
MB;BS (Ib), MPH (Field Epid) (Ib), Ph.D. (Copenhagen), FMCPH (Nig)

**A. T. Salawu, Lecturer II**
MB;BS (Ib), M.Sc. (Epid) (Ib); M.B.A. (Ib)

MEDICAL STATISTICS

**Oyindamola B. Yusuf, Reader**
B.Sc. (Ib.), M.Sc. (Ib.) Ph.D. (Ib.) Cstat (UK.)

**O. M. Akpa, Senior Lecturer**
B.Sc. (Ilorin), M.Sc. (Ilorin), Cert. (India), Ph.D. (Ilorin)

**A. S. Adebowale, Senior Lecturer**
B.Sc. (Ado), P.G.D. (Lagos), M.Sc. (Lagos), M.Sc. (Ife), Ph.D. (Ife)

**J. O. Akinyemi, Senior Lecturer**
B. Tech (Akure), M.Sc. (Ib.), Ph.D. (Ib.)

**A. F. Fagbamigbe, Lecturer I**
B.Sc. (Ilorin), M.Sc. (Lancaster) Ph.D (Ib.)

**B. M. Gbadebo, Lecturer I**
B.Sc. (Ife), M.Sc. (Ife) Ph.D (!fe)

**R. F. Afolabi, Lecturer I**
B.Sc. (Ilorin), M.Sc. (Ilorin). Ph.D (Ilorin)

ADJUNCT LECTURERS

**O. Ayeni, Adjunct Professor**
B.Sc. (Ib.), M.Sc., Med, Stat, (Lond.), Ph.D,(Lond.)

**M. C. Asuzu, Professor**
M.B.B.S (Ib.) D.O.H.&S., M.Sc. (Mc. Master), F. M. C. P. H. (Nig.)


21 September, 2020

Dear Editor,

**PLOS ONE: Your submission PONE-D-20-04600R2 - [EMID:9e65145cf6089ba0]**

Thank you for this great observation in the process of reviewing our paper titled "**Semi-parametric model for timing of first childbirth after HIV diagnosis among women of childbearing age in Ibadan, Nigeria**". This concern is greatly appreciated.

We have provided a statemet to address consent in Methods section, lines 103-104 of the revised manuscript to read as follows -

"Anyone who selected a "Yes" was enrolled to participate in the study **after written informed consent was obtained**".

We hope it meets your expectation.

Thank you.

Yours sincerely,

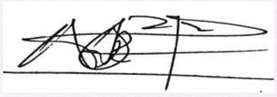


Rotimi F. Afolabi
Mobile telephone: +234-806-607-5196
Email address: rotimifelix@yahoo.com
