# Supplementary material for: Semi-parametric model for timing of first childbirth after HIV diagnosis among women of childbearing age in Ibadan, Nigeria
Source: PLoS One. 2020 Oct 7;15(10):e0240247. doi: 10.1371/journal.pone.0240247 (PMC7540879; doi:10.1371/journal.pone.0240247)
Supplement: Attachment — Submitted filename: Response to Reviewers.docx [file pone.0240247.s001.docx]

16 July, 2020

Dear Editor,

**Cover Letter: Revision required [PONE-D-20-04600] - [EMID:1d35fbf60d84f59a]**

Thank you for the accelerated review of our paper titled “**Semi-parametric model for time to first birth after HIV diagnosis among women in South-West, Nigeria**”. The comments are valuable in terms of enriching the quality of the paper.

Data are available upon request to researchers who meet the criteria for access to confidential data guided by our institutional policies. Formal request can be sent to our Institutional Review Board ([uiuchec@gmail.com](mailto:uiuchec@gmail.com)).

Please find the responses to the editorial and reviewer’s comments below for your information and attention.

Regards!

Yours sincerely,

Rotimi F. Afolabi

Mobile telephone: +234-806-607-5196

Email address: [rotimifelix@yahoo.com](mailto:rotimifelix@yahoo.com)

**Reviewers Notes and Responses**

| **S/N** | **Comments** | **Authors’ reply and Amendment Made** |
| --- | --- | --- |
| I | **Editorial comment - Journal Requirements** | |
| 1. | Please ensure that your manuscript meets PLOS ONE's style requirements, including those for file naming. The PLOS ONE style templates can be found at https://journals.plos.org/plosone/s/file?id=wjVg/PLOSOne_formatting_sample_main_body.pdf and https://journals.plos.org/plosone/s/file?id=ba62/PLOSOne_formatting_sample_title_authors_affiliations.pdf | We appreciate these comments. This has been addressed with the following modification in the revised manuscript. |
| 2. | We suggest you thoroughly copyedit your manuscript for language usage, spelling, and grammar.  Upon resubmission, please provide the following:   - The name of the colleague or the details of the professional service that edited your manuscript - A copy of your manuscript showing your changes by either highlighting them or using track changes (uploaded as a *supporting information* file) - A clean copy of the edited manuscript (uploaded as the new *manuscript* file) | This very important suggestion by the reviewer is appreciated. We have edited its grammar accordingly, and we hope that all grammar issues have been well addressed.   - The name of the colleague that edited our manuscript is Dr Ayo Stephen Adebowale - A tracked copy of the manuscript showing our changes is tagged Revised Manuscript with Track Changes and uploaded as a supplementary file - A clean copy of the edited manuscript is uploaded as the new *manuscript* file |
| 3. | We note that you have indicated that data from this study are available upon request. PLOS only allows data to be available upon request if there are legal or ethical restrictions on sharing data publicly. For more information on unacceptable data access restrictions, please see http://journals.plos.org/plosone/s/data-availability#locunacceptable-  data-access-restrictions.  In your revised cover letter, please address the following prompts:  a) If there are ethical or legal restrictions on sharing a de-identified data set, please explain them in detail (e.g., data contain potentially sensitive information, data are owned  by a third-party organization, etc.) and who has imposed them (e.g., an ethics committee). Please also provide contact information for a data access committee, ethics committee, or other institutional body to which data requests may be sent.  b) If there are no restrictions, please upload the minimal anonymized data set necessary to replicate your study findings as either Supporting Information files or to a stable,  public repository and provide us with the relevant URLs, DOIs, or accession numbers. For a list of acceptable repositories, please see http://journals.plos.org/plosone/s/dataavailability#  loc-recommended-repositories.  We will update your Data Availability statement on your behalf to reflect the information you provide. | This comment is noted, and this have been addressed in the cover letter. The data are avaivalable on request subject to ethical and institutional policies. Data enquiries can be sent to uiuchec@gmail.com.  Thank you. |
| 4. | Thank you for stating the following in the Acknowledgments Section of your manuscript:  'The project from which this data was extracted was supported by the Medical Education Partnership Initiative in Nigeria (MEPIN) project funded by Fogarty International Centre, Office of AIDS Research, and the National Human Genome Research Institute of the National Institute of Health, the Health Resources and Services Administration (HRSA) and the Office of the U.S. Global AIDS Coordinator under Award Number R24TW008878.   - We note that you have provided funding information that is not currently declared in your Funding Statement. However, funding information should not appear in the Acknowledgments section or other areas of your manuscript. We will only publish funding information present in the Funding Statement section of the online submission form. - Please remove any funding-related text from the manuscript and let us know how you would like to update your Funding Statement. Currently, your Funding Statement reads - as follows: - 'The author(s) received no specific funding for this work.' | This is well received and has been addressed in the revised manuscript.  The funding statement included is now:  “The authors received no specific funding for this present work. However, the project described in this study was supported by the Medical Education Partnership Initiative in Nigeria (MEPIN) funded by Fogarty International Centre, the Office of AIDS Research, and the National Human Genome Research Institute of the National Institute of Health, the Health Resources and Services Administration (HRSA) and the Office of the U.S. Global AIDS Coordinator under Award Number R24TW008878. The content is solely the responsibility of the authors and does not necessarily represent the official views of the funding organisation” |
| II | **Reviewer #1: Comments** |  |
| 1. | The title should be modified as per the study objectives | Thank you for this comment and recommendation. We have modified the title and it now reads: “Semi-parametric model for timing of first childbirth after HIV diagnosis among women of childbearing age in Ibadan, Nigeria” |
| 2. | The abstract is too long (particularly, result part). Try to make it short and precise | This very important observation is highly appreciated, and we have shortened the result section of the abstract accordingly. |
| 3. | Some edition is needed. (eg: line 28, 32 pronoun “we” is wrongly used). | We sincerely apologize for the mistake. We have made the necessary correction and adjustment in the revised manuscript. |
| 4. | How much of the data were 'valid' and what was the level of missingness for the variables of interest that you used? | The level of missingness was very low (less than 3%) because data collection was supervised to ensure completeness |
| 5. | Be consistent in your references citation (eg. Line 74-line 78 different reference citation ) | This very important comment is appreciated. We have revised and reflected the Vancouver (bracket) reference style as appropriate in the revised version of the manuscript. |
| 6. | On Line 124: you have stated that “women aged 18-49 years who had had at least a child-birth…. ” were considered in the study. From line 129-30 it has been stated that “For proper event history analysis, women who did not have a child birth after HIV diagnosis were censored…. ” which contradicts the former statement. Take a look. | We thank the reviewer for this observation!  For the sake of clarity, we have provided the statement “women aged 18-49 years who had had at least a child-birth before or after HIV diagnosis, …”  Please refer to the   - **Method, Study population and variables subsection, page 5, line 116**   for the necessary correction and modification in the revised manuscript clean copy |
| 7. | Line 211: why you need to have exponential distribution test? | We appreciate this comment!  Indeed, the exponential distribution test is the authors information in order to guide the choice of either parametric or otherwise statistical method. However, based on this comment, we have excluded the exponential distribution test in the revised version of the manuscript. |
| 8. | Line 245: Employment status (p=0.049) violated the proportional hazard assumption but the graph for employment status (Fig 2) is parallel which implied that PH assumption is not violated. How could it be? | We appreciate the reviewer for this important observation. However, it is important to note that the subjective interpretation of the graphical method of assessing proportional hazard assumption has necessitated the call for formal goodness-of-fit test asides the graphical method (Ata & Sozer, 2007; In & Lee, 2019). Even though the use of two approaches simultaneously is recommended owing to the problems listed above, Schoenfeld residual test is more objective than an log-log survival plot.  However, the inclusion of clinically important variables has resolve this challenge in the revised version of the manuscript. |
| 9. | Table 2 on page 13: It has been identified that Cox PH model is not appropriate since PH assumption is violated for some explanatory variables. Once the violation of PH assumption is detected, no need to keep cox PH model for comparison purpose with extended cox model. It is better to consider only the extended model and interpret the result. Model comparison should be made (using AIC, BIC, ) only between appropriate models for the particular data. | Special thanks to the reviewer for this observation and the authors equally share the concerns of the reviewer.  Based on the observations, Table 2 has been restructured and modified.  Please kindly refer to:   - **Results section, Table 2**   for the changes and modifications provided in the revised manuscript. |
| 10. | Interpretation of HR is not clear. | We have revised the statenment interpreting the HR in the revised manuscript.  Please kindly refer to:   - **Methods section, page 9, lines 191-192**   for the changes and modifications provided in the revised manuscript-clean copy |
| 11. | Be consistent when you write the response variable (first birth interval after diagnosis or time to first birth after diagnosis). Line 337 “first birth after HIV diagnosis” is not good expression | This has been addressed in the revised version of the document as suggested.  kindly refer to:   - **Conclusion section, page 16, lines 328-329**   for the changes and modifications provided in the revised manuscript-clean copy.  Thank you. |
| 12. | Inclusion exclusion criteria are not clearly stated. | We appreciate these comments of the reviewer. This has been addressed with the following statements added in the revised manuscript  “…before or after HIV diagnosis …” for clarity on inclusion criteria  “All critically ill clients who were not well enough to provide responses were excluded” for the exclusion criterion  Kindly refer to **Method section, page 5, lines 116– 118** of the revised manuscript (clean copy) for the details |
| 13. | Limitation should be separated from discussion | The comment has been attended to in the revised document. Thank you |
| **II** | **Reviewer #2: Comments** | |
| 1. | Generally the authors need to check on grammatical mistakes that abound in the manuscript. Some have been highlighted. | We appreciate the reviewer for this important observation. Editorial review has been carried out on the document and we also sought the assistance of expert in this regard.  Please refer to the revised version of the manuscript for further detail of corrections and modifications. |
| 2. | Data Analysis   - Kaplan Meier.   The notations and definitions are misleading. The authors use the random variable T to denote a woman who had her FBI_HIV beyond t time. Normally T denotes the future lifetime random variable of an individual aged 0(newborn) .In this context, T could be time to first birth after HIV diagnosis, so that s(t0 is the probability of birth after t years.   - Equation 2 is wrong, the integral should be from 0 to t and not 1.   Following the author’s arguments, the probable definition of f(t) is the probability that a woman has a child at time t.   - The equations are poorly written, kindly use latex, Microsoft equation etc. - The author is also silent on the censoring mechanism assumed in the data. - Line 160: Mixed up notations: What does t_i represent? Survival times, or failure times/birth times. There is also a general mix up under this heading. | We agree totally with these comments of the reviewer.   - We regret the mix-up. Based on these comments, we have effected the changes in the revised version of the manuscript as appropriate. - The equation 2 has been corrected and modified in the revised version of the manuscript - Mathtype equation has been used to rewrite the equations - We have also included right censored in the revised manuscript. Kindly refer to **Methods section, page 6, line 122**.   Please see the revised manuscript for the corrections and modification effected. |
| 3. | COX PH   - Line 190, 191: Not clear   Women Characteristics   - Although the author states that table 1 displays among others the Kaplan Meier survival estimates, I have been unable to see the Kaplan Meier survival estimates. | This comment by the reviewer is noted. For the sake of clarity, the method section has been modified to address the issue raised.  Please kindly refer to:   - **Method section, Data analysis subsection, lines 185-187**   for the detailed modification in the revised manuscript  Median survival estimates as presented in Table 1 is a product of Kaplan Meier survival estimation. |
| 4. | Results and discussions   - It would be of great value for the authors to outline how they selected the covariates. What feature selection techniques were employed? - Important features like use of ART and status of the partner have been left out. | This very important observation by the reviewer is appreciated.  Although we did not provide much detail in the method about the issues raised, we have included the statement “All significant variables (p<=0.25) based on the log-rank test including clinically important varibles were included in extended Cox regression model ” in the revised manuscript.  kindly refer to:   - **Methods section, Data analysis subsection, page 9, lines 194-196**   The initial choice of selection of variables based on p<0.05 for log-rank test and simple Cox PH significance, use of ART was insignificant. However, using the revised p<=0.25 for selection of variables at bivariate stage, use of ART duration including the desire for more chidren remained significant at the multivariate level.  Please refer to:   - **Discussion section, paragraphs 4 and 6, page 16**   for further statements provided in the revised manuscript |
| III | **Additional corrections based on authors’ consideration** | Based on our critical considerations, we have made the following corrections.   1. We have provided the statement “using STATA 14 SE (StataCorp LP, College Station, USA)”   Kindly refer to  **Methods section, page 9, line 198** in the revised manuscript   2. We have provided overall survival and hazard functions, tagged Fig 1, to describe women’s first birth interval after HIV diagnosis   Kindly refer to  **Results section, Fig 1** in the revised manuscript   3. We re-produce log-log survival plot due to the inclusion of duration of ART use and desire for more children, tagged Fig 2   Kindly refer to  **Results section, Fig 2** in the revised manuscript  **Thank you.** |
